# Effect of Tea Tree Essential Oil on the Quality, Antioxidant Activity, and Microbiological Safety of Lightly Processed Lily (*Lilium brownii* var. *viridulum*) during Storage

**DOI:** 10.3390/foods13132106

**Published:** 2024-07-02

**Authors:** Yuge Guan, Sainan Lu, Yan Sun, Rentao Zhang, Xinghua Lu, Linjiang Pang, Lei Wang

**Affiliations:** 1 School of Food and Health, Zhejiang Agricultural and Forestry University, Hangzhou 311300, China; 2 Stoddart Institute of Molecular Science, Department of Chemistry, Zhejiang University, Hangzhou 310027, China

**Keywords:** lily, tea tree essential oils, antioxidant activity, nutrient, reactive oxygen species

## Abstract

The Lanzhou lily is a regionally distinctive vegetable; the emergence of lightly processed lilies has addressed the inconvenience of consuming fresh lilies. However, the cleaning and impurity removal during the processing of lightly processed lily may strip off its original protective barrier and affect the edible quality. As one of the preservation methods, tea tree essential oil (TTEO) has the characteristics of being green, safe, and efficient preservative properties. This study focused on investigating the effects of different concentrations (25 μL/L, 50 μL/L, and 100 μL/L) of TTEO on the quality and microbiological safety of lightly processed lily. The results showed that compared with the control, appropriate concentrations of TTEO treatment could delay weight loss, improve appearance, firmness, and sensory quality, and maintain microbiological safety with the best effect observed at 50 μL/L. Meanwhile, TTEO treatment induced phenylalanine ammonia-lyase activity, thereby increasing the total phenolic content. Furthermore, TTEO enhanced the superoxide dismutase (SOD) and ascorbate peroxidase (APX) activity, which reduced O_2_-· production rate and H_2_O_2_ content. TTEO inhibited lipoxygenase (LOX) activity, reducing the relative conductivity and malondialdehyde content, thereby delaying lipid peroxidation and quality deterioration. This indicates that TTEO could enhance antioxidant capacity by regulating reactive oxygen species (ROS) metabolism and delay the quality deterioration of lightly processed lily by inhibiting lipid peroxidation.

## 1. Introduction

Lilies (*Lilium brownii* var. *viridulum*), belonging to the Liliaceae family and Lilium genus, are highly valued not only for their nutritional and unique flavor but also for their medicinal and health-promoting properties [1]. Grown in Lanzhou, Gansu, lilies boast superior production and quality, with an annual output value reaching CNY 2.5 billion [2]. However, the cleaning and peeling process of lily scales before cooking has posed an inconvenience for consumers. In recent years, with the improvement of living standards and the enhancement of health awareness, the lightly processed (or fresh-cut) fruit and vegetable industry has experienced rapid development. Lightly processed fruits and vegetables, characterized by convenience such as ready-to-eat, ready-to-use, and ready-to-cook, have gained popularity among consumers [3]. Therefore, processing lilies into lightly processed scales, after cleaning and peeling, has become increasingly favored by consumers. The emergence of the lightly processed lily industry not only effectively addresses the cumbersome pre-processing steps before consuming lilies but also reduces urban waste through the comprehensive utilization of by-products generated from pre-processing, aligning with the concepts of environmental protection and sustainable development in the modern food processing industry [4]. During the processing of lilies, unavoidable wounding stress disrupts the physiological metabolic balance of the organism, accelerating water loss, and nutrient degradation, and providing favorable conditions for microbial growth [5]. Especially, the wounding stress caused by cutting during the processing differs from the localized mechanical stress typically experienced by plants. Lightly processed fruits and vegetables not only lose metabolic coordination of the whole fruit but also sustain irreparable, systemic injuries, triggering the rapid generation of reactive oxygen species (ROS) within a short period [6]. Appropriate concentrations of ROS can serve as signaling molecules to participate in the defense response of fruits and vegetables against wounding stress, enhancing the resistance of fresh-cut products [7]. However, excessive accumulation of ROS disrupts the metabolic balance in plants, increases the burden of ROS, causes severe oxidative damage, and then reduces the antioxidant activity of fresh-cut products [8]. Additionally, wounding stress induces ethylene production and wound respiration, leading to the disintegration and senescence of lightly processed tissues. Moreover, it disrupts the localized distribution of enzymes and substrates, triggering changes such as cell wall degradation, and phenolic and lipid oxidation, resulting in increased enzymatic browning of tissues, which leads to the destruction of cell membrane integrity, generation of off-flavors, formation of secondary metabolites, and callus tissue formation [9]. After undergoing light processing, the original protective system of lilies is disrupted, leading to a series of physiological and biochemical changes that greatly affect the appearance, nutritional content, and antioxidant activity of fresh-cut products, severely restricting the development of the lightly processed lily industry. There are some studies about the preservation methods for controlling the deterioration of lightly processed lily bulbs. For example, Huang et al. found that UV-C treatment of 4.5 kJ m^−2^ can increase the activity of phenylalanine ammonia-lyase (PAL), thereby increasing the total phenol content and reducing polyphenol oxidase (PPO) and peroxidase (POD), delaying the degree of browning in lightly processed lilies [1]. Subsequently, researchers confirmed the significant role of key enzymes such as lipoxygenase (LOX) and critical metabolites such as malondialdehyde (MDA) in the browning process of lightly processed lilies [10,11]. Recently, Li et al. revealed from a transcriptional level that modified atmosphere treatment maintains the integrity of lily cell membranes by inhibiting lipid peroxidation, thereby effectively preserving their quality [12]. In addition, researchers found that sweet orange essential oil could effectively inhibit the microbial growth of lightly processed lilies during storage [13]. Overall, previous studies on the changes in quality during the storage of lightly processed lilies mainly focused on controlling enzymatic browning. However, enzymatic browning occurs as a reparative stress response to mechanical injury in plants, which is jointly regulated by factors such as ROS signaling molecules and antioxidant systems. Currently, the mechanism of ROS regulation in response to lightly processed stress-induced changes in lily quality remains unclear. Therefore, in-depth research on ROS metabolism during the post-harvest storage of lightly processed lily bulbs not only addresses key factors restricting the quality of processed lilies but also improves their added value and economic benefits, providing new insights for researching relevant preservation and regulation technologies.

Plant essential oils have become a research hotspot in recent years for their safety, environmental friendliness, and efficiency in preserving fruits and vegetables. As one kind of essential oil, tea tree essential oil (TTEO) is a natural antibacterial agent and the few essential oils included in the European Pharmacopoeia, and it is also listed in the monograph of the European Medicines Agency [14,15]. In recent years, researchers have begun to explore its effects on preserving fruits and vegetables. Ce et al. found that TTEO could change the permeability of cell membranes and disrupt their integrity, leading to the leakage of substances such as electrolytes, proteins, and DNA inside the cells, thereby damaging microbial cells and inhibiting their growth [16]. Due to its excellent antibacterial properties, TTEO can also be used as a preservative in the food industry. For example, Wei et al. studied the pre-harvest spraying of TTEO on strawberry fruits, which showed that spraying TTEO can slow down the loss of fruit hardness, delay fruit aging, effectively control post-harvest rot of strawberries by reducing microbial load at harvest, induce defense responses, reducing H_2_O_2_ accumulation by increasing catalase (CAT) and ascorbate peroxidase (APX) enzyme activities, and increasing PAL enzyme activity to accumulate more phenolic substances in strawberry fruits [17]. However, the impact of TTEO on the microbial safety of lightly processed lilies has not been reported, and the concentration of TTEO used is uncertain, limiting its application in the lightly processed lily industry. This study used Lanzhou lilies as experimental materials and employed exogenous TTEO treatment to dynamically monitor the physiological and biochemical qualities of lightly processed lilies, including their appearance, sensory evaluation, hardness, etc.

This study aimed to determine the optimal concentration of TTEO treatment that effectively maintains the edible quality of lightly processed lilies. Additionally, the study investigated the changes in ROS content and antioxidant substances (total phenols and vitamin C) during the storage of lightly processed lilies and explored the relationship between the activities of antioxidant enzymes, and antioxidant capacity, which further elucidates the mechanism by TTEO effectively preserve the quality at the physiological and biochemical levels. This study lays the theoretical foundation for the development of new preservation technologies for lightly processed fruits and vegetables.

## 2. Materials and Methods

### 2.1. Tea Tree Essential Oil and Reagents

Tea tree essential oil (TTEO) was purchased from Ji’an Zhongxiang Natural Plant Co., Ltd. (Ji’an, China). Catechol, guaiacol, DPPH, and ABTS were purchased from Aladdin Co., Ltd. (Shanghai, China). All other chemical reagents used were analytical grade and purchased from Tianjin Kemio Chemical Reagent Co., Ltd. (Tianjin, China).

### 2.2. Plant Material and Pretreatment

Lily bulbs (*Lilium brownii* var. *viridulum*) were obtained from Qilihe District, Lanzhou City, Gansu Province. Mature, disease-free, mechanically undamaged, and uniformly sized six-year-old fresh samples were selected for this experiment. The lily bulb samples were disinfected in 0.2 mL/L sodium hypochlorite solution for 5 min, and washed with distilled water, then, samples were put on a ventilator to dry naturally for 2 h. The 150 g lily bulb samples were treated with 0, 25, 50, and 100 μL/L tea tree essential oil (TTEO) vapor in 4 L closed containers at 4 °C for 24 h, respectively. After fumigation treatment, each 75 g samples were placed in a polyethylene (PE) plastic tray covered with PE preservative film. All samples were stored at 4 °C for 18 days, and the relevant parameters of each group sample were measured every 3 days.

### 2.3. Sensory Evaluation and Appearance Quality Assessment

The sensory evaluation (color, odor, texture, freshness, decay, overall quality) was assessed using a 5-point scale: 5 points for no change; 4 points for slight change; 3 points for moderate change; 2 points for significant change; 1 point for severe change [18]. The sensory evaluation results of all treatment groups’ samples at the end of storage (18th day). The whiteness index (WI) was used to evaluate the color of the sample, it was calculated as the following: WI = 100 − [(100 − L*)^2^ + (a*)^2^ + (b*)^2^]^1/2^, where L*, a*, b* represents lightness, redness and yellowness, respectively, these indexes were evaluated by using a CR400 colorimeter (Konica Minolta Inc., Tokyo, Japan). Meanwhile, the changes in appearance quality of the processed lilies during the entire storage period were documented with photographs.

### 2.4. Determination of Respiration Rate, Weight Loss Rate, and Firmness

Lilies were placed in a 500 mL airtight container with a rubber stopper for 30 min. The respiration rate was performed using an F-940 gas analyzer (F-940 STORE II, Felix, CA, USA) [19]. The result of respiration rate was calculated as the following formula, where C_1_ represents the volume fraction of CO_2_ in sample (%); C_2_ represents the volume fraction of CO_2_ in the atmosphere (%); V represents the volume (mL); m represents the mass of sample (kg); t represents the respiration time (h).

$$\mathrm{respiration}\;\mathrm{rate}=\frac{\left(C_{1}-C_{2}\right)\;÷\;100\;\times \;V}{m\times t}\;\times \;1.96$$

The weight of sample is measured daily, and the weight loss rate during storage is calculated using the mass loss method [20].

The firmness of lily is assessed by using texture profile analysis (TPA) with a TA. XT Plus texture analyzer (TA-XT plus, Stable Micro Systems, Surrey, UK) [21]. During the testing process, a cylindrical probe with a diameter of 5 mm at a speed of 1 mm/s.

### 2.5. Determination of Nutrient Content

#### 2.5.1. Determination of Starch and Reducing Sugar Content

The starch content was determined by using the 3,5-dinitrosalicylic acid method [22]. Briefly, 1 g of sample was mixed with 10 mL of 80% ethanol solution, after stirring, the mixture was extracted at 80 °C for 30 min. After cooling, the filtrate was collected, and the residue was re-extracted twice. The filtrates were combined and diluted to 100 mL as the extract solution for starch content determination. Similarly, the reduced sugar content was determined using the same method after extracting the sample with distilled water at 80 °C for 30 min [23].

#### 2.5.2. Determination of Soluble Protein Content

The soluble protein content was determined using the Coomassie brilliant blue method [24]. A 1 g amount of sample was mixed with 5 mL of distilled water, centrifuged at 12,000 rpm at 4 °C for 20 min, and the supernatant was collected. Then, 1 mL of the supernatant was mixed with 5 mL of Coomassie brilliant blue G-250 solution, and the absorbance was measured at 595 nm after 2 min of standing. The soluble protein content in the sample was calculated based on the standard curve of bovine serum albumin (y = 0.0031x + 0.2651) (100–400 μg/mL), and the result was expressed as mg/g of fresh weight.

#### 2.5.3. Determination of Vitamin C (Vc) Content

The Vc content was determined by using colorimetric method [25]. A 1 g amount of sample was mixed with 5 mL of oxalic acid solution (0.05 M) containing 0.2 mM EDTA, centrifuged at 13,000 rpm at 4 °C for 20 min, and the supernatant was collected. Then, the reaction system consisted of 2 mL supernatant, 2 mL oxalic acid–EDTA, 0.5 mL metaphosphate acetic acid, 1 mL 5% sulfuric acid, and 2 mL 5% ammonium molybdate solution, which was measured at 760 nm, and the Vc content was calculated based on the standard curve (y = 0.1653x − 0.0047) (0–100 mg/L), the result was expressed as mg/g of fresh weight.

#### 2.5.4. Determination of Total Phenolic Content

The total phenolic content was determined according to the method described by Dai et al. [21]. The phenolic extraction was used with 80% methanol containing 1% (*v*/*v*) HCl, and the extraction solution mixed with Folin–Ciocalteu reagent, which was measured the absorbance at 760 nm. The total phenolic content was calculated based on the standard curve of gallic acid at concentration range from 0 to 50 mg/L, (y = 0.0064x + 0.0158).

### 2.6. Determination of ROS Content

The content of hydrogen peroxide (H_2_O_2_) and the production rate of superoxide anion O_2_-·) were determined using commercial assay kits (H_2_O_2_-1-Y and SA-1-G) following the manufacturer’s instructions (Shanghai Lianmai Co. Ltd., Shanghai, China). The absorbance was measured at specific wavelengths using a microplate reader (Multiskan GO, Thermo Scientific, Waltham, MA, USA), and the results were expressed as μmol/g for H_2_O_2_ content and nmol/g·min for the production rate of O_2_-·, respectively

### 2.7. Determination of Electrolyte Leakage

The measurement method of electrolyte leakage was according to the previous report with slight modifications [26]. Specifically, three uniformly sized circular slices of lily bulbs were taken using a puncher. After rinsing, the slices were air-dried and then mixed with 20 mL of distilled water for 1 h at 35 °C. The initial conductivity (E_0_) and final conductivity (E_1_) were measured using a conductivity meter [27]. Subsequently, the conical flask was heated in a water bath for 0.5 h, and the conductivity (E_2_) was measured again. The formula for calculating cell membrane permeability is as follows: cell membrane permeability (%) = (E_1_ − E_0_)/E_2_ × 100%.

### 2.8. Determination of Malondialdehyde (MDA) Content and Lipoxygenase (LOX) Activity

The MDA content of samples was determined by using the thiobarbituric acid (TBA) colorimetric method [28,29,30]. Briefly, after extraction with 10% TBA, the solution was centrifuged at 4 °C and 10,000 rpm for 20 min, 2 mL of the supernatant was taken and mixed with 2 mL of 0.67% TBA. The mixture was then reacted in a water bath for 20 min, and the absorbance was measured at 600 nm, 450 nm, and 532 nm. The result of MDA content was expressed as μmol/kg of fresh weight.

The determination of LOX activity was performed according to the protocol provided by the LOX-1-W kit (Suzhou Keming Biological Technology Co., Ltd., Suzhou, China). The enzyme activity was calculated based on the absorbance at 280 nm. One unit of LOX activity was defined as the catalytic absorbance change of 0.01 per minute per gram. The results are expressed as U/g.

### 2.9. Determination of Antioxidant Enzyme Activity

The CAT enzyme was extracted with sodium phosphate (0.1 mol/L, pH 6.4), and then the supernatant was centrifugated at 12,000 rpm for 30 min [31]. For CAT assay, 3 mL of 20 mmol/L H_2_O_2_ solution and 0.5 mL of enzyme extract were mixed, and the absorbance change at 240 nm was monitored for 180 s.

The extraction method for POD was the same as that for CAT enzyme. In brief, 2 mL of 0.05 mol/L guaiacol mixed with 0.5 mL of enzyme solution for 5 min at 30 °C. Then, 1 mL of 0.08% H_2_O_2_ was added, and the absorbance at 470 nm was scanned for 1 min.

The SOD activity was determined using the SOD-1-Y assay kit (Suzhou Kaiming Biological Co., Ltd., Suzhou, China). The absorbance of the reaction system was measured at 560 nm, and one unit of SOD activity was defined as the amount of enzyme required to inhibit 50% of the reaction system.

The APX activity was determined according to the method described by Zhou et al. [21]. The enzyme was extracted using 0.1 mol/L potassium phosphate (pH 7.5). The reaction system consisted of 2.6 mL reaction buffer (containing 0.1 mmol/L EDTA and 0.5 mmol/L ascorbic acid), 0.1 mL of enzyme extract, and 0.3 mL of 2 mmol/L H_2_O_2_ solution. The absorbance change at 290 nm was monitored. The results of CAT, POD, SOD, and APX activity were expressed as units per gram of sample (U/g).

### 2.10. Determination of Antioxidant Activity

The present study used DPPH and ABTS radical scavenging assays to evaluate the antioxidant activity of lightly processed lilies. Specifically, 5 g of sample was mixed with 20 mL of 70% ethanol solution and homogenized. The mixture was then subjected to extraction by ultrasonic cleaner (KQ-100E, Kunshan Ultrasound Instrument Co., Ltd., Kunshan, China) continuously at 40 °C for 40 min, followed by centrifugation at 4 °C and 10,000 rpm for 20 min. Next, 100 μL of the supernatant was transferred to a 96-well plate and mixed with 100 μL of DPPH solution for measurement. The DPPH radical scavenging capacity was calculated using the formula: (A_0_ − A_t_)/A_0_, where A_0_ represents the absorbance of the ethanol blank control, and A_t_ represents the absorbance of the sample.

For the determination of the ABTS radical scavenging ability, 20 μL of the sample was mixed with 80 μL of ABTS solution and then measured the absorbance at 734 nm. The measurement procedure involved oscillation for 15 s, followed by incubation at 20 °C for 6 min [32]. The result was calculated using the formula: (A_0_ − A_t_)/A_0_, where A_0_ represents the absorbance of the ethanol blank control and A_t_ represents the absorbance of the sample.

### 2.11. Total Plate Counts, Molds, and Yeasts Content Determination

Samples were homogenized and diluted, and then plated onto different agar media for microbial count determination, including plate count agar (PCA) for total viable count, violet red bile dextrose agar (VRBDA) for coliform count, and potato dextrose agar (PDA) for mold and yeast count. The plates were incubated at 37 °C for 24 h, and the colonies were counted using a colony counter [33]. The results of total plate counts, molds, and yeast content were expressed as Log CFU/g.

### 2.12. Data Analysis

All experiments were conducted in triplicate, and the results were expressed as the mean ± standard error. Statistical analysis was performed using SPSS 25.0 software. One-way analysis of variance (ANOVA) followed by LSD multiple comparisons was used to determine the significant differences among groups (*p* < 0.05). Different letters in the results indicate significant differences between treatment groups (*p* < 0.05).

## 3. Results and Discussion

### 3.1. Quality-Related Parameters of Lightly Processed Lily

#### 3.1.1. Appearance and Sensory Evaluation

**Figure 1:**
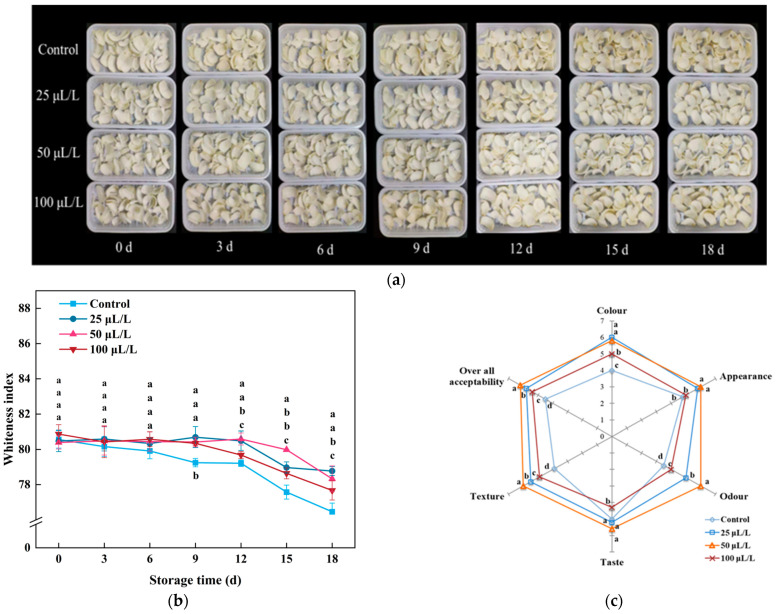
Effects of TTEO treatment on the appearance color (**a**), whiteness index (WI) (**b**), and sensory evaluation (**c**) of minimally processed lily bulbs during storage. Different letters on the same day represented a significant difference among treatment factors (*p* < 0.05).

The visual changes in processed fruits and vegetables are the most intuitive indicators for assessing the degree of quality deterioration during storage, enabling consumers to judge the quality of samples [34]. As shown in Figure 1a, the control group samples exhibited slight browning on 3rd day, while the lightly processed Lily bulbs treated with 25 and 50 μL/L TTEO displayed characteristics of thick and white texture, which showed no significant differences in appearance quality compared to the original sample. By the 9th day of storage, the control group samples showed significant browning, attributed to direct exposure to air leading to moisture evaporation and discoloration [35]. The WI and sensory evaluation results of all treatment groups’ samples (Figure 1b,c) indicate that the treatment with 50 μL/L TTEO showed the highest levels, followed by 25 μL/L and 100 μL/L, consistent with the results of appearance quality. In summary, fumigation treatment with TTEO could effectively maintain the color of lily bulbs, thereby preserving their appearance quality and sensory quality, with the concentrations of 50 μL/L and 25 μL/L exhibiting better preservation effects on the color and appearance of lightly processed lily bulbs.

#### 3.1.2. Respiration Rate, Weight Loss Rate, Firmness

As the most critical life activity of processed fruits and vegetables, respiration not only provides energy for life activities but also serves as the central hub for the synthesis and metabolism of organic substances within tissues [36]. As shown in Table 1, the respiration of lightly processed Lily bulbs decreased throughout the entire storage period. On the 18th day of storage, the respiration of the control group, 25 μL/L, 50 μL/L, and 100 μL/L TTEO-treated groups decreased by 26.08%, 34.01%, 35.52%, and 17.65%, respectively, compared to the 0th day of storage. This may be due to the severe mechanical damage to the tissues of Lily bulbs after light processing, resulting in a gradual reduction in metabolism, disrupting the metabolic balance of respiration, thereby reducing respiration [37]. Unlike cabbage, pumpkin, and other vegetables, Lily bulbs did not exhibit a respiration peak during storage, which may be because Lily belongs to non-respiratory climacteric vegetables [38]. Additionally, this study found that appropriate concentrations of TTEO (25 μL/L and 50 μL/L) treatment significantly reduced the respiration intensity of lightly processed Lily bulbs in the late stage of storage.

The weight loss rate is an important indicator for evaluating the freshness and post-harvest preservation effect of fruits and vegetables. As shown in Table 1, the processed treatment significantly increased the weight loss rate of Lily bulbs, mainly because the metabolic activities of processed fruits and vegetables require a large amount of substrate consumption, and the internal moisture of the organism will also transpire to the outside of the organism, thereby increasing the weight loss rate [39]. The weight loss rates of the 25 μL/L and 50 μL/L TTEO treatment groups’ samples were significantly (*p* < 0.05) lower than those of the control group throughout the entire storage period. On the 18th day of storage, the weight loss rates of the 25 μL/L and 50 μL/L TTEO groups were 25.21% and 25.07%, respectively, which was lower than those of the control group. The regulatory effect of TTEO on weight loss rate is similar to that of respiration. Therefore, it is inferred that TTEO could effectively maintain the weight loss rate by reducing the respiration and the consumption of substrates and moisture inside the organism, thereby effectively maintaining the weight loss rate of the product.

Firmness is a standard of judging the acceptability of fresh-cut fruits and vegetables, directly affecting consumers’ acceptance of the product [40]. As shown in Table 1, the firmness of lily bulbs in all groups decreased significantly (*p* < 0.05) during storage; this may be due to reasons such as tissue dehydration and aging and then resulted in the softening of lightly processed lily bulbs. TTEO treatment significantly delayed the decrease in firmness, showing better effects in the late storage period. Different concentrations of TTEO had different effects on firmness. After 9 days, the treatment with 50 μL/L TTEO showed the best effect, followed by 100 μL/L TTEO. In terms of comprehensive physical and chemical quality, only 50 μL/L TTEO could simultaneously maintain firmness, weight loss rate, and respiration intensity, while other concentrations of firmness only exhibit effects in a single aspect, indicating that the selection of appropriate concentration treatment with firmness as a preservation method is particularly important for the preservation of lightly processed fruits and vegetables.

#### 3.1.3. Nutrient Content

Lily bulbs contain abundant starch, imparting their crispy texture, which serves as the main energy source for lily bulbs’ physiological metabolism [1]. As shown in Table 1, the starch content of samples in all treatment groups continuously decreased during storage from 0 to 18 days. Especially in the control group, the starch content sharply declined in the first 6 days of storage, possibly due to the increasing physiological metabolism caused by severe damage to the organism, and then accelerating the hydrolysis of starch in lily bulbs [41]. However, the rate of starch decline in the TTEO treatment groups was relatively slower, especially in the 25 μL/L and 50 μL/L TTEO groups. At 18 days, the starch content was 29.61% and 31.83% higher, respectively, compared to the control group.

In contrast to the change trend in starch content, the reduced sugar content of lightly processed lily bulbs fluctuated during storage, with the reduced sugar content of the control group consistently higher than that of the TTEO treatment groups (Table 1). The reduced sugar content increased sharply and peaked on the 12th day of storage, followed by a sharp decrease from 12 to 15 days. However, the changes in reducing sugar content were relatively stable in TTEO treatment groups, remaining around 8.0%. This indicates that TTEO effectively regulates lily bulbs’ physiological metabolism, slowing down the hydrolysis of starch and the accumulation of reducing sugars, thereby delaying fruit aging [42].

Soluble protein is a vital nutrient in fruits and vegetables, closely related to the cell’s water retention capacity and providing protection to the cell’s vital substances and biological membranes [43]. As shown in Table 1, the soluble protein content of the control group samples generally increased first and then decreased during storage, especially experiencing a sharp decline after 9 d of storage, from 0.86 mg/g to 0.43 mg/g. This may be attributed to the severe mechanical damage to lily bulbs after light processing, disrupting the balance of physiological metabolism and leading to the degradation of enzymes and bound proteins in tissues [44]. It is worth noting that the soluble protein level of 50 μL/L TTEO treatment groups was highest in all groups during the whole storage, which was 62.79% higher than the control group. This may be due to the conversion of some soluble sugars into proteins, resulting in an increase in soluble protein content [45]. These results indicate that an appropriate concentration of TTEO has a positive effect on lily bulbs’ metabolism, slowing down the degradation rates of starch and soluble proteins, thereby maintaining the nutrient content.

### 3.2. ROS Content

**Figure 2:**
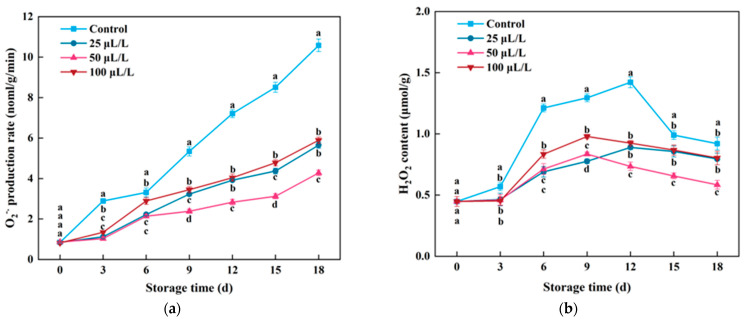
Effects of TTEO treatment on the O_2_-·production rate (**a**) and H_2_O_2_ content (**b**) of lightly processed lily bulbs during storage. Different letters on the same day represented a significant difference among treatment factors (*p* < 0.05).

ROS, as by-products of normal oxygen metabolism in fruits and vegetables, mainly include O_2_-· and H_2_O_2_. Excessive accumulation of ROS could induce oxidative damage to tissues, reflecting the severity of oxidative stress [46]. As shown in Figure 2a, with the extension of storage time, the O_2_-·production rate increased continuously in all treatment groups. Among them, the O_2_-· production rate in the control group samples increased the fastest, reaching 12.45 times that of before storage at the end of storage. This may be because fresh fruits and vegetables undergo wounding stress during light processing, different from the localized mechanical damage stress experienced by general plants. Lightly processed fruits and vegetables not only lose the metabolic coordination of the whole fruit and vegetable but also suffer irreparable, overall damage without self-protection. Additionally, cell tissues are completely exposed to air, leading to a rapid induction of ROS [47]. TTEO treatment delayed the increase in the production rate of O_2_-·, with the treatment effect of 50 μL/L being better than that of 25 μL/L and 100 μL/L. At the end of storage, the production rate of O_2_-· in the 50 μL/L treatment group was 6.30 nmol/min/g lower than that in the control group.

The H_2_O_2_ content of lightly processed lily showed an increasing and then decreasing trend with the extension of storage time (Figure 2b). The H_2_O_2_ content of the control and 25 μL/L TTEO-treated sample reached its maximum on the 12th day of storage, increasing by 2.18 times and 0.64 times, respectively, compared to the levels of the original sample. However, the H_2_O_2_ content of the 50 μL/L and 100 μL/L groups began to decrease from the 9th day of storage. At the end of storage (18th day), the 50 μL/L TTEO treatment effectively inhibited the accumulation of H_2_O_2_ content, which was 35.18% lower than that of the control group. The inconsistent changes in O_2_-· and H_2_O_2_ levels in lightly processed Lily bulbs may be due to the complex antioxidant enzyme system in plants, which can regulate ROS levels according to changes in fruit and vegetable physiological metabolism to maintain the balance of ROS metabolism in tissues [48]. These results indicate that 50 μL/L TTEO treatment can effectively reduce O_2_-· production rate and H_2_O_2_ content, similar to the previous report that plant essential oil treatment (sweet orange EO) could decrease the ROS content, and then reduce oxidative damage to tissue cells, and prolong the shelf life of lily bulbs [49].

### 3.3. Key Metabolites and Enzyme Activity in the Pathway of Membrane Lipid Peroxidation

**Figure 3:**
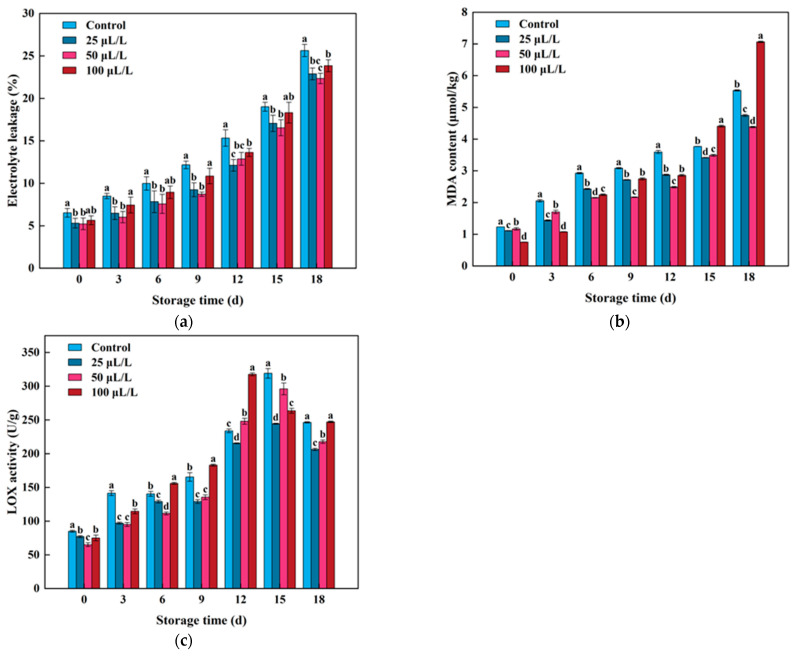
Effects of TTEO treatment on the electrical conductivity (**a**), MDA content (**b**), and LOX activity (**c**) of lightly processed lily during storage. Different letters on the same day represented a significant difference among treatment factors (*p* < 0.05).

The wounding stress during the light processing could directly lead to the rupture of tissue cell membranes, cause the exudation of cytoplasm, and further result in the membrane lipids peroxidation reactions. In general, electrical conductivity and MDA content can be used to evaluate the degree of membrane lipid oxidation in fresh-cut fruits and vegetable tissues. As shown in Figure 3a, the electrical conductivity of samples in all treatment groups continuously increased, with the electrical conductivity of TTEO treatment groups being lower than that of the control group throughout the entire storage period. Among them, the electrical conductivity of the 50 μL/L group was at its lowest level in the late storage period, being 12.97% and 12.87% lower than that of the control group on the 15th and 18th days of storage, respectively. Similarly, the MDA content increased gradually with the extension of storage time, consistent with the trend in electrical conductivity. In the early storage period, TTEO treatment significantly decreased MDA content (Figure 3b). However, unlike electrical conductivity, in the late storage period (15th and 18th days), the MDA content of the high-concentration TTEO treatment group (100 μL/L) was significantly higher than that of the control group, possibly because the higher concentration of essential oil inducing LOX activity (Figure 3c), thereby accelerating the membrane lipid peroxidation reaction of lightly processed lily bulbs and leading to the accumulation of MDA.

The increase in LOX activity after light processing of lily bulbs may be a response of plant tissues to external injury stress, accompanied by the rapid accumulation of MDA, causing significant damage to fruit and vegetable cells and inducing the deterioration of fresh-cut fruit quality. This phenomenon is consistent with the findings in kiwi [24], fresh-cut potatoes [45], fresh-cut mushrooms [50], and fresh-cut apples [51]. The previous study found that thyme essential oil and sweet orange essential oil treatment could effectively regulate their respiratory pathways and physiological metabolism activities, reduce the accumulation of MDA, and decrease the degree of cell membrane damage and membrane lipid peroxidation, thereby maintaining the integrity of cell membrane structure and compartmentalization and delaying fruit senescence and death of lightly processed Lily bulbs [49]. However, the study in fresh-cut lettuce found that treatment with marjoram essential oil increased the degree of membrane lipid peroxidation, which indicates that different types of plant essential oils have different regulatory mechanisms on membrane lipid peroxidation [52]. Therefore, when selecting plant essential oils for the preservation of lightly processed products, it is necessary to consider not only the treatment concentration of plant essential oils but also the characteristics of essential oils themselves to develop new types of preservatives.

### 3.4. Vitamin C Content and APX Activity

**Figure 4:**
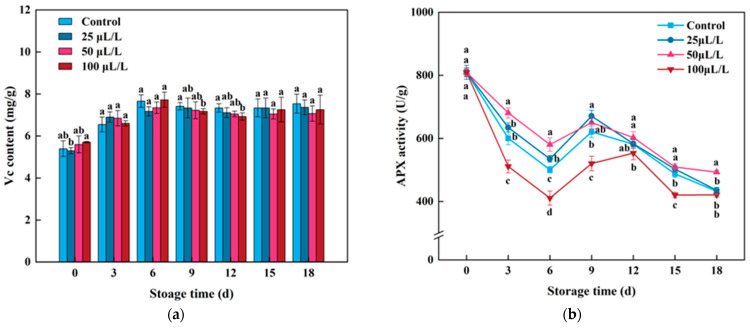
Effects of TTEOs treatment on the Vc content (**a**) and APX activity (**b**) of minimally processed lily bulbs during storage. Different letters on the same day represented a significant difference among treatment factors (*p* < 0.05).

Vitamin C (Vc) is one of the essential antioxidant components, playing a crucial role in antioxidant defense mechanisms in fruits and vegetables. As shown in Figure 4a, the Vc content in Lily bulbs was 5.40 mg/g at the original storage time. After light processing, the Vc content gradually increased with the extension of storage time, reaching a peak on the 6th day, followed by a stable trend. The initial increase in Vc content may be a response of lightly processed Lily bulbs to mechanical damage, while the subsequent stabilization may be due to irreversible tissue damage and aging, resulting in a weakened response to stress [53]. Throughout the storage period, TTEO treatment did not show a significant differential effect on the Vc content in lightly processed lily bulbs.

**Figure 5:**
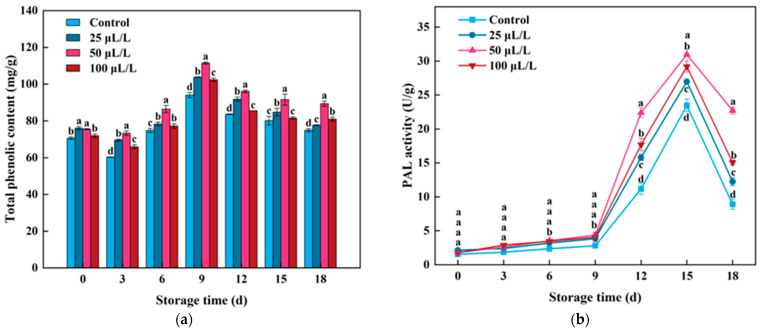
Effects of TTEO treatment on the total phenolic content (**a**) and PAL activity (**b**) of lightly processed lily during storage. Different letters on the same day represented a significant difference among treatment factors (*p* < 0.05).

Ascorbate peroxidase (APX) enzyme is a key enzyme in oxidizing Vc to dehydroascorbate and reducing H_2_O_2_, effectively eliminating free radicals in cells, and thereby enhancing the resistance of plant tissues [30]. As shown in Figure 5a, both the control group and the TTEO-treated groups exhibited a fluctuating trend of APX activity with increasing storage time, showing a decrease first, followed by an increase, and then a decrease again. Among them, the APX activity in 25 μL/L and 50 μL/L TTEO treatment groups was consistently higher than that in the control group throughout the storage period. However, the significant difference in APX activity between the TTEO treatment group and the control group was not sustained throughout the entire storage period, and this result was consistent with the effect of TTEO treatment on the Vc content in lily bulbs. This may be because Vc acts as a catalytic substrate for APX, synergistically exerting antioxidant effects through the Vc-GSH cycle [29]. Therefore, the regulatory effect of TTEO on APX activity and Vc content in Lily bulbs is consistent. The APX activity in the 100 μL/L TTEO treatment group was lower than that in the control group, possibly due to the high concentration of essential oil treatment causing cell damage in Lily bulbs, accelerating tissue aging, and reducing enzyme activity [54].

### 3.5. Total Phenolic Content and PAL Enzyme Activity

As another major source of antioxidants in lily bulbs, phenolic compounds are important indicators for evaluating the antioxidant quality of fruits and vegetables. As shown in Figure 5a, the total phenolic content of lightly processed lily bulbs showed a fluctuating trend of first decreasing, then increasing and then decreasing again. The initial decrease in total phenolic content within the first 3 days of storage may be a response of lightly processed lily bulbs to mechanical damage, with phenolic compounds being used to resist a large amount of ROS induced by fresh-cut processing, thereby accelerating the oxidation rate of phenolic compounds and leading to a decrease in their content. After 3 days of storage, the total phenolic content increased, reaching its maximum on the 9th day of storage, with all groups showing an increase, with the control group being 33.22% higher than the initial value. This result indicates that fresh-cut processing induced the accumulation of phenolic compounds, consistent with previous studies in fresh-cut eggplant [55], fresh-cut carrots [56], and fresh-cut dragon fruit [7], demonstrating that fresh-cut processing increases the total phenolic content of fresh-cut products. Similarly, on the 9th day of storage, the 25 μL/L, 50 μL/L, and 100 μL/L TTEO treatment groups increased by 36.46%, 47.62%, and 42.35%, respectively, compared to the initial values, and the total phenolic content in the TTEO groups was significantly higher than that in the control group. This result indicates that tea tree essential oil treatment can significantly increase the total phenolic content of lightly processed lily bulbs.

Phenylalanine ammonia-lyase (PAL) is the first critical limiting enzyme in the phenylpropanoid pathway, responsible for catalyzing the conversion of phenylalanine to cinnamic acid, which then participates in the synthesis of phenolic substances in plants, protecting plants from oxidative stress damage [57]. As shown in Figure 5b, PAL activity showed a trend of first increasing, then decreasing during storage. Within 18 days of storage, PAL activity peaked on the 15th day, with the control group, 25 μL/L, 50 μL/L, and 100 μL/L TTEO groups increasing by 15.14, 12.64, 17.20, and 16.68 times, respectively, compared to the original levels. This result indicates that storage time significantly affects the PAL activity in lightly processed lily bulbs, consistent with similar results reported in fresh-cut dragon fruit [58]. In addition, TTEO treatment at different concentrations significantly increased the PAL activity in lightly processed lily bulbs, with the increase rate being 50 μL/L > 100 μL/L > 25 μL/L, indicating that the 50 μL/L TTEO treatment had the best effect on enhancing PAL activity. This result is consistent with the trend of changes in total phenolic content, indicating that TTEO treatment may stimulate the phenylpropanoid metabolism process by increasing PAL activity, thereby accelerating the synthesis of phenolic compounds in lightly processed Lily bulbs [25].

### 3.6. Antioxidant Enzyme Activity

**Figure 6:**
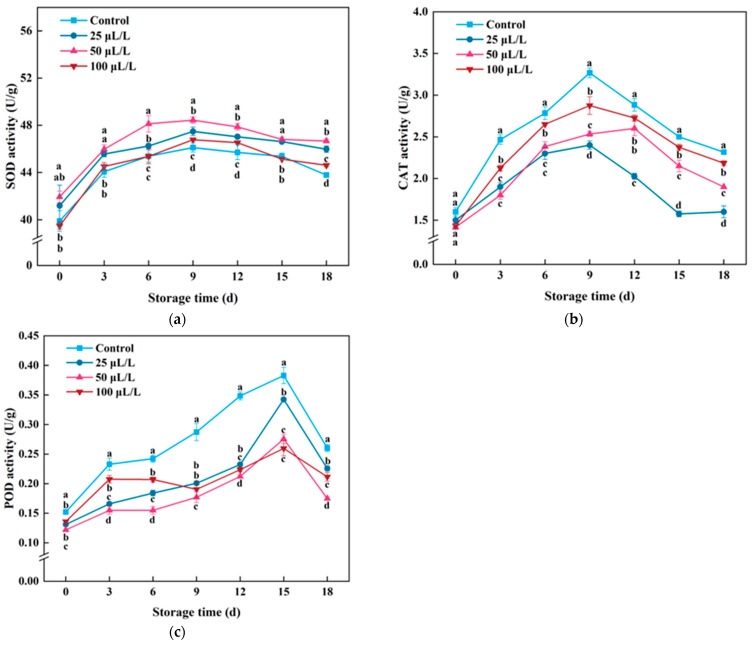
Effects of TTEO treatment on the SOD activity (**a**), CAT activity (**b**), and POD activity (**c**) of lightly processed lily during storage. Different letters on the same day represented a significant difference among treatment factors (*p* < 0.05).

Superoxide dismutase (SOD) is a crucial antioxidant enzyme, and it catalyzes the dismutation of O_2_-· into O_2_ and H_2_O_2_, playing a vital role in the oxidation and antioxidant balance in plant organisms [53]. As shown in Figure 6a, SOD activity gradually increased with the extension of storage time. On the 9th day of storage, the SOD activity of each group reached its peak, with the control group, 25 μL/L, 50 μL/L, and 100 μL/L TTEO-treated groups increasing by 15.30–18.52% compared to the initial value. However, after 9 days of storage, the SOD activity of each treatment group gradually decreased, possibly due to irreversible oxidative damage to tissue cells with increasing storage time, leading to a decrease in SOD enzyme activity [59]. Throughout the storage period, the 50 μL/L TTEO group showed relatively stable activity, with overall activity higher than other treatment groups, which is consistent with the study in strawberries, indicating that appropriate concentration of TTEO treatment can increase the SOD enzyme activity of fruits and vegetables [15]. However, there was no significant difference between the 100 μL/L TTEO group and the control group. Combining the above results, it can be concluded that 50 μL/L TTEO can significantly increase the SOD activity of lightly processed lily bulbs, thereby reducing the rate of O_2_-· release, slowing down the oxidation process of fresh-cut tissues, and better maintaining the quality of the samples.

Catalase (CAT) is another oxidative enzyme that can remove reactive oxygen species. It mainly catalyzes the decomposition of H_2_O_2_ into O_2_ and H_2_O, thereby playing a role in scavenging free radicals and protecting organisms from oxidative damage [60]. As shown in Figure 6b, during the storage period, the CAT activity in all groups was increased first and then decreased with the prolonged storage time, which reached its peak on the 9th day. However, the CAT activity of lily bulbs in the control group was significantly (*p* < 0.05) higher than that in the TTEO-treated group during the storage period. However, in the late stage of storage, the CAT activity gradually decreased, indicating that the ability of lightly processed lily bulb tissues to remove ROS decreased, the oxidative damage to tissues increased, and the tissue repair ability decreased, which may lead to a deeper degree of browning of lightly processed lily bulbs [61]. In this experiment, although the CAT activity of the TTEO treatment group was lower than that of the control group, the rate of H_2_O_2_ content was also lower than that of the control group, indicating that the pathway for lightly processed lily bulbs to decompose H_2_O_2_ is not solely dependent on CAT enzyme but may be related to non-enzymatic antioxidant systems such as phenolic compounds [62]. That is, TTEO may inhibit the generation of H_2_O_2_ by increasing the ability of non-enzymatic antioxidant systems. The results of this study are inconsistent with those of fresh-cut cabbage and fresh-cut broccoli, indicating that the mechanism of ROS removal in fresh-cut fruits and vegetables under different stress conditions may vary, especially the significant wounding stress caused by fresh-cut fruits and vegetables differs from the localized mechanical stress experienced by typical plants. Fresh-cut fruits and vegetables not only lose the metabolic coordination of the entire fruit or plant, but also suffer irreparable, self-protective systemic injuries, and the cellular tissue is in direct contact with air, which can rapidly induce the outbreak of ROS [63].

Peroxidase (POD) is also a widely distributed heme metal enzyme in plants with high redox activity, which can remove H_2_O_2_ to alleviate the damage of ROS to cell membranes, exerting its antioxidant effect and increasing the resistance of fresh-cut fruits and vegetables to mechanical damage stress [47]. As shown in Figure 6c, the POD activity of all groups first increased and then decreased, consistent with the changes in POD activity in pear fruits during storage reported by Fan et al. [64]. indicating that fresh-cut processing induced the POD activity in the early storage period, and then enhanced the resistance in lightly processed lily bulbs. However, the POD activity of the TTEO treatment group was significantly (*p* < 0.05) lower than that of the control group, consistent with the degree of browning of lightly processed lily bulbs. This may be because POD has dual biological functions. It not only decomposes free radicals, but also plays an important role in the oxidative browning process of plant tissues, oxidizing phenolic substances into quinone substances using O_2_ produced during plant metabolism, and then catalyzing a series of reactions such as dehydration and polymerization to form brown–black substances, thus catalyzing the oxidative browning process [21,65]. The results of this study demonstrate that the POD activity of lily bulbs is closely related to the degree of browning, and TTEO treatment may delay tissue browning by inhibiting POD activity.

### 3.7. Antioxidant Capacity

**Figure 7:**
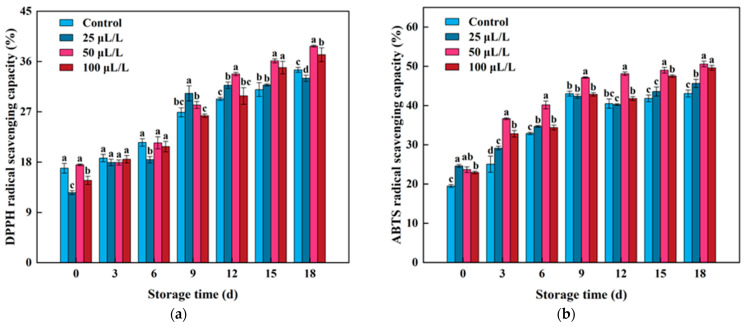
Effects of TTEOs treatment on DPPH radical scavenging capacity (**a**) and ABTS radical scavenging capacity (**b**) of lightly processed lily during storage. Different letters on the same day represented a significant difference among treatment factors (*p* < 0.05).

In this study, DPPH and ABTS radical scavenging capacities were selected to evaluate the antioxidant activity of lightly processed lily bulbs. As shown in the figures (Figure 7a), with the increase in storage time, the DPPH radical scavenging capacity of lightly processed lily bulbs in each group gradually increased. From the 12th to 15th day of storage, the DPPH scavenging capacity of lightly processed lily bulbs treated with different concentrations of TTEO was higher than that of the control group. At the end of storage, the 50 μL/L and 100 μL/L treatment groups were 12.32% and 7.83% higher than the control group, respectively, while the DPPH scavenging capacity of the low-concentration group (25 μL/L) was 4.38% lower than that of the control group. This result indicates that selecting an appropriate concentration of TTEO can enhance the antioxidant capacity of lightly processed lily bulbs, while improper concentration treatment may have the opposite effect. A study on fresh-cut cantaloupe similarly showed that improper concentration of oil treatment can cause damage to cell tissues, resulting in severe membrane lipid damage and adversely affecting product quality [66].

Like DPPH, ABTS is also a very stable free radical. ABTS can be oxidized by K_2_S_2_O_8_ to generate blue–green radical cations ABTS+, which can be cleared by antioxidants with hydrogen supply capability, thereby maintaining the antioxidant capacity of the sample itself. As shown in Figure 7b, the ABTS radical scavenging capacity of lightly processed lily bulbs gradually increased with the extension of storage time. It is worth noting that the maximum ABTS radical scavenging capacity of the control group, 25 μL/L, 50 μL/L, and 100 μL/L TTEO-treated groups reached 43.02%, 42.37%, 50.57%, and 49.62%, respectively. This result indicates that the 50 μL/L TTEO-treated group achieved the best improvement effect in ABTS radical scavenging capacity. Similarly, 50 μL/L TTEO also showed good performance in enhancing DPPH radical scavenging capacity, while the effect of 25 μL/L TTEO treatment on antioxidant capacity is not significant in lightly processed Lily bulbs. The reason for this result is that 50 μL/L TTEO provides strong free radical scavenging capacity, showing good clearing effects, while 25 μL/L TTEO may not provide sufficient concentration to effectively clear free radicals [67]. The 100 μL/L TTEO may be because the concentration of essential oil is too high, affecting the integrity of lily bulb tissues, resulting in lower antioxidant capacity throughout the storage period [16]. Therefore, 50 μL/L TTEO treatment had the best performance in maintaining antioxidant capacity.

### 3.8. Microbial Survival

**Figure 8:**
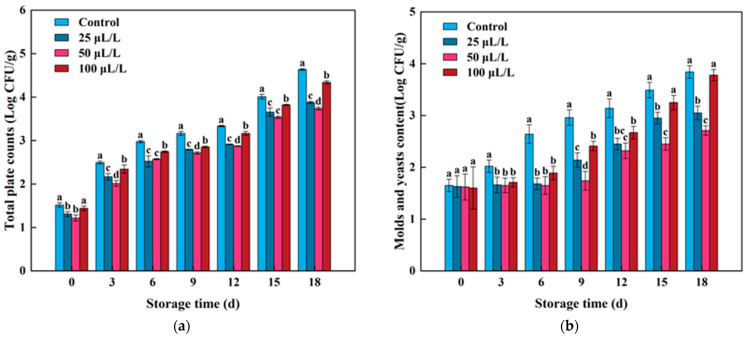
Effects of TTEOs treatment on total plate counts (**a**) and molds and yeasts (**b**) of minimally processed lily bulbs during storage. Different letters on the same day represented a significant difference among treatment factors (*p* < 0.05).

For lightly processed fruits and vegetables, the leakage of juice caused by mechanical cutting creates an environment with exposed cytoplasm, high water activity, suitable pH, and abundant sources of nutrients on the surface of the product, providing favorable conditions for microbial growth and reproduction, which further affect the microbial safety [68]. To test the microbial safety of lightly processed lily bulbs during storage, this study tested the total bacterial counts, molds, and yeast content. As shown in Figure 8a, the total bacterial counts of all samples increased continuously with the extension of storage time. Overall, except for 0 days, the total bacterial counts in the TTEO treatment group were significantly lower than that in the control group at other storage times (*p* < 0.05). At the end of storage, the total bacterial counts in the 50 μL/L TTEO-treated group were reduced by 19.4% compared to the control group, indicating that TTEO has a good antibacterial effect on lightly processed lily bulbs, which may be mainly due to the antibacterial effects of substances such as terpenes and cineole in the essential oil [69]. However, the antibacterial mechanism of TTEO is not yet clear, and future research can use the main components of TTEO to treat lightly processed lily bulbs and analyze in detail its effects on the quantity of different types of microorganisms, thereby elucidating the antibacterial mechanism.

As shown in Figure 8b, molds and yeast content in lily bulbs of each treatment group showed varying degrees of growth throughout the storage period, with the TTEO treatment group consistently lower than the control group. Among them, the 50 μL/L TTEO has significant potential for inhibiting the growth of molds and yeasts, with molds and yeasts counts 1.13 log CFU/g lower than the control group after 18 days of storage. In addition, on the 18th day of storage, the molds and yeasts content in the 25 μL/L and 100 μL/L TTEO treatments were 0.79 log CFU/g and 0.06 log CFU/g lower than the control group, respectively. This result is inconsistent with the study on fresh-cut cantaloupe [66], which showed that the higher the oil concentration, the better the antibacterial effect. In this study, it was found that there is no linear relationship between the concentration of TTEO and the microbial count in lightly processed lily bulbs. The reason for this may be that the TTEO used in this study not only acts through its own antibacterial properties but may also induce the synthesis of more secondary metabolites such as phenolic compounds, thereby exerting better antibacterial properties [1]. This study also confirms the regulation of TTEO on the quality and microbial is the result of the interaction of multiple metabolic pathways. Therefore, choosing the appropriate concentration of essential oil treatment is crucial for the preservation effect of fresh-cut fruits and vegetables.

## 4. Conclusions

We present the first study comparing the effects of TTEO (0, 25, 50, 100 μL/L) treatment on the quality, antioxidant activity, and microbiological safety of lightly processed lily. The result indicated that 50 μL/L TTEO treatment effectively maintains the appearance, sensory quality, starch content, soluble protein content, and physiological qualities such as firmness, weight loss rate, and respiration intensity of lightly processed lily bulbs. Additionally, 50 μL/L TTEO treatment could reduce the cell membrane permeability and MDA content by reducing POD and LOX activity and then resulted in the delaying lipid peroxidation processes in lily bulbs. Meanwhile, treatment with 50 μL/L TTEO effectively increases PAL activity, thereby leading to a 47.62% increase in phenolic compound content. The 50 μL/L TTEO treatment increases SOD enzyme activity and results in the lower O_2_-· production rate and the H_2_O_2_ content (decreasing by 35.18% than control). This comprehensive approach led to a substantial improvement in DPPH and ABTS scavenging capacity, with a remarkable increase of 12.32% and 17.55%, respectively, compared to the control. Furthermore, treatment with 50 μL/L TTEO has great potential for inhibiting microbial growth during storage of lightly processed lily bulbs, the total bacterial count in the 50 μL/L TTEO treatment group is reduced by 19.4%, and the mold and yeast counts are 1.13 log CFU/g lower than the control group. Therefore, treatment with 50 μL/L TTEO is an effective method for preserving physiological, and biochemical quality, microbial safety, and then enhancing the antioxidant capacity of lightly processed lily bulbs.

## Figures and Tables

**Table 1:** Effects of TTEO treatment on quality indexes of lightly processed lily.

Storage (d)	Treatment	Respiration Rate (mg/(kg·h))	Weight Loss Rate (%)	Firmness (N)	Starch Content (%)	Reducing Sugar Content (%)	Soluble Protein Content (mg/g)
0	Control	36.92 ± 1.01 b	0.00	3.11 ± 0.16 a	23.51 ± 0.32 a	8.20 ± 0.05 a	0.75 ± 0.03 a
	25 μL/L	38.66 ± 0.38 a	0.00	3.16 ± 0.06 a	23.32 ± 0.19 a	8.19 ± 0.07 a	0.72 ± 0.04 a
	50 μL/L	36.66 ± 0.28 b	0.00	3.22 ± 0.07 a	23.44 ± 0.21 a	8.23 ± 0.06 a	0.75 ± 0.02 a
	100 μL/L	34.23 ± 0.03 c	0.00	3.23 ± 0.03 a	23.50 ± 0.17 a	8.16 ± 0.10 a	0.73 ± 0.03 a
3	Control	31.83 ± 0.41 b	1.03 ± 0.16 b	2.87 ± 0.08 b	21.97 ± 0.16 b	8.26 ± 0.11 a	0.70 ± 0.22 ab
	25 μL/L	35.23 ± 2.02 a	1.03 ± 0.09 b	2.87 ± 0.04 b	23.21 ± 0.09 a	8.18 ± 0.04 a	0.71 ± 0.03 ab
	50 μL/L	34.36 ± 1.20 a	1.05 ± 0.18 b	3.01 ± 0.07 a	23.42 ± 0.23 a	8.15 ± 0.07 a	0.73 ± 0.02 a
	100 μL/L	35.03 ± 0.72 a	1.60 ± 0.14 a	3.06 ± 0.03 a	23.09 ± 0.17 a	8.12 ± 0.05 a	0.68 ± 0.01 b
6	Control	31.54 ± 1.05 b	2.60 ± 0.12 a	2.78 ± 0.01 b	19.11 ± 0.15 b	8.65 ± 0.13 a	0.78 ± 0.02 b
	25 μL/L	33.15 ± 0.53 a	2.08 ± 0.01 b	2.78 ± 0.05 b	23.12 ± 0.16 a	8.07 ± 0.09 b	0.84 ± 0.03 a
	50 μL/L	31.11 ± 0.53 b	1.96 ± 0.24 b	2.93 ± 0.04 a	23.09 ± 0.23 a	8.02 ± 0.12 b	0.85 ± 0.02 a
	100 μL/L	32.07 ± 0.72 a	2.53 ± 0.27 a	2.96 ± 0.03 a	22.73 ± 0.29 a	8.10 ± 0.11 b	0.72 ± 0.01 c
9	Control	27.35 ± 1.15 a	3.23 ± 0.22 a	2.63 ± 0.04 c	18.87 ± 0.09 c	9.58 ± 0.12 a	0.86 ± 0.01 b
	25 μL/L	27.03 ± 1.28 a	2.73 ± 0.02 b	2.77 ± 0.03 b	23.11 ± 0.12 a	7.65 ± 0.06 c	0.87 ± 0.02 b
	50 μL/L	26.91 ± 1.72 a	2.66 ± 0.33 b	2.90 ± 0.05 a	22.98 ± 0.14 a	7.73 ± 0.05 bc	0.93 ± 0.02 a
	100 μL/L	25.63 ± 0.46 b	3.66 ± 0.36 a	2.80 ± 0.01 b	22.16 ± 0.17 b	7.92 ± 0.11 b	0.82 ± 0.01 c
12	Control	27.51 ± 0.60 a	4.71 ± 0.10 a	2.53 ± 0.02 c	18.53 ± 0.20 c	13.51 ± 0.09 a	0.63 ± 0.02 c
	25 μL/L	26.07 ± 0.52 b	3.66 ± 0.03 b	2.61 ± 0.03 b	22.92 ± 0.19 a	8.09 ± 0.12 b	0.79 ± 0.04 b
	50 μL/L	26.03 ± 0.79 b	3.54 ± 0.32 b	2.70 ± 0.05 a	22.73 ± 0.08 a	8.15 ± 0.14 b	0.89 ± 0.05 a
	100 μL/L	26.10 ± 1.27 a	4.54 ± 0.44 a	2.67 ± 0.10 a	21.55 ± 0.12 b	8.32 ± 0.10 b	0.57 ± 0.02 d
15	Control	23.80 ± 0.07 b	5.77 ± 0.07 a	2.50 ± 0.04 b	18.01 ± 0.13 c	11.47 ± 0.14 a	0.59 ± 0.02 c
	25 μL/L	21.95 ± 0.78 c	4.05 ± 0.17 b	2.60 ± 0.09 a	22.78 ± 0.21 a	7.99 ± 0.05 c	0.65 ± 0.03 b
	50 μL/L	23.24 ± 1.35 b	4.22 ± 0.29 b	2.67 ± 0.03 a	22.59 ± 0.15 a	7.84 ± 0.06 c	0.73 ± 0.02 a
	100 μL/L	28.05 ± 1.35 a	5.50 ± 0.35 a	2.63 ± 0.04 a	21.64 ± 0.11 b	8.25 ± 0.09 b	0.51 ± 0.01 d
18	Control	27.29 ± 0.98 a	7.10 ± 0.23 a	2.39 ± 0.02 c	17.12 ± 0.16 c	13.81 ± 0.15 a	0.43 ± 0.01 b
	25 μL/L	25.51 ± 0.17 b	5.31 ± 0.22 b	2.49 ± 0.01 b	22.19 ± 0.17 a	7.76 ± 0.07 c	0.65 ± 0.02 a
	50 μL/L	23.64 ± 0.17 c	5.32 ± 0.19 b	2.64 ± 0.03 a	22.57 ± 0.23 a	7.85 ± 0.05 c	0.70 ± 0.03 a
	100 μL/L	28.19 ± 0.12 a	7.19 ± 0.35 a	2.62 ± 0.02 a	21.41 ± 0.19 b	8.69 ± 0.06 b	0.39 ± 0.01 c

Note: Different lowercase letters indicate significant differences between the groups (*p* < 0.05) by LSD multiple comparisons.

## Data Availability

The original contributions presented in the study are included in the article, further inquiries can be directed to the corresponding author.
